# Supplementary material for: A Global Screen for Assembly State Changes of the Mitotic Proteome by SEC-SWATH-MS
Source: Cell Syst. 2020 Feb 26;10(2):133–155.e6. doi: 10.1016/j.cels.2020.01.001 (PMC7042714; doi:10.1016/j.cels.2020.01.001)

P56378 | 68MP_HUMAN | MP68 C14orf2 PRO1574
Monomer MW [kDa]: 6.662 Monomer expected elution fraction: 64

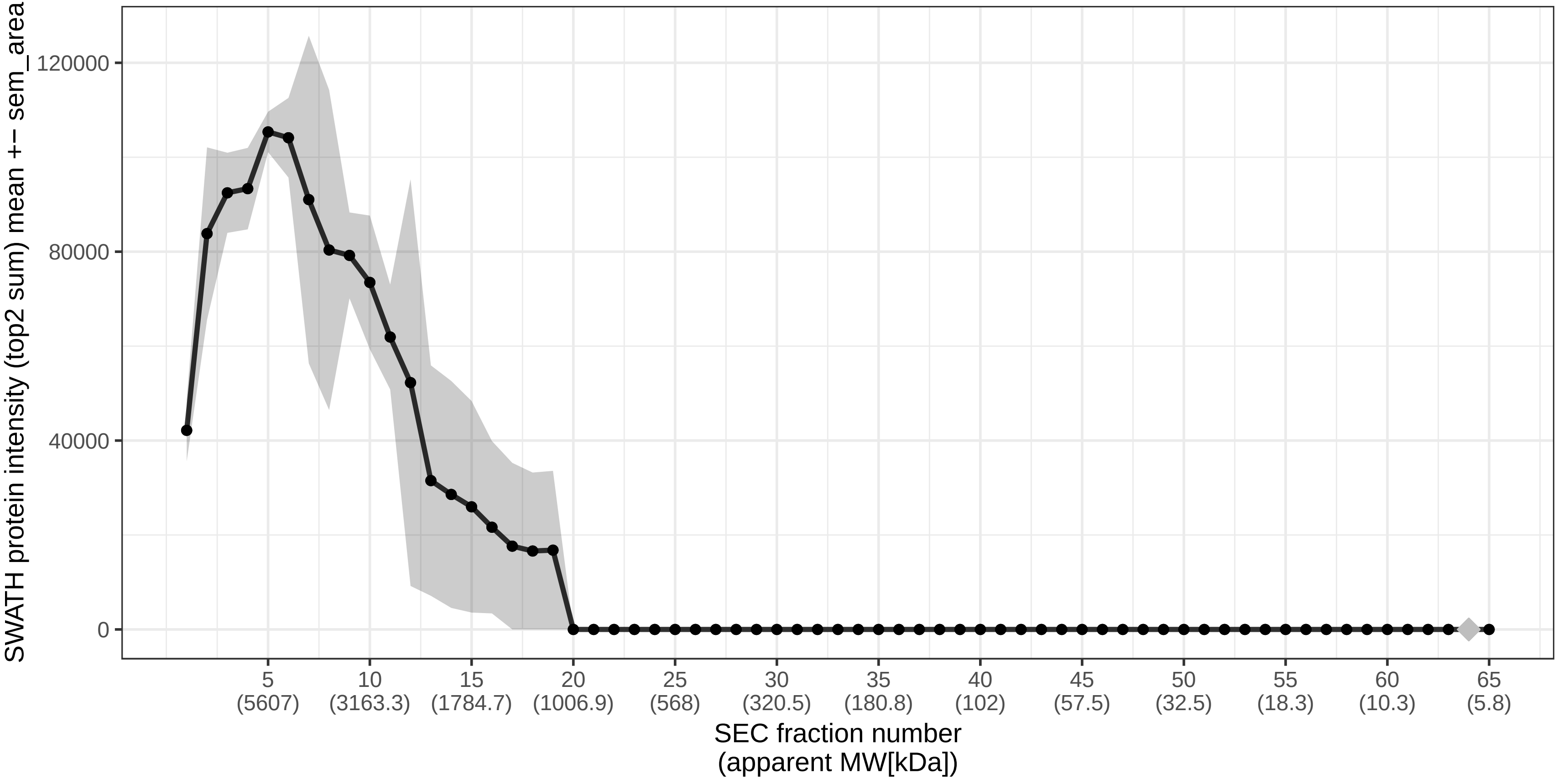

Supplement: Data S1. SEC-SWATH-MS Protein Chromatograms, Related to Figure 1 [file mmc6.zip › SECchrom_P56378_68MP_HUMAN_MP68_C14orf2_PRO1574.pdf]
